# Supplementary material for: Broad anti-sarbecovirus responses elicited by a single administration of mosaic-8 RBD-nanoparticle vaccine prepared using atomic layer deposition
Source: iScience. 2025 Sep 25;28(11):113649. doi: 10.1016/j.isci.2025.113649 (PMC12549395; doi:10.1016/j.isci.2025.113649)
Supplement: Document S1. Figures S1–S4 [file mmc1.pdf]

iScience, Volume *28*

Supplemental information

Broad anti-sarbecovirus responses elicited by a single administration of mosaic-8 RBD-nanoparticle vaccine prepared using atomic layer deposition

Alexander A. Cohen, Jennifer R. Keeffe, Annie V. Rorick, Semi Rho, Ange-Célia Priso Fils, Lusineh Manasyan, Han Gao, Priyanthi N.P. Gnanapragasam, Hans H. Funke, Theodore W. Randolph, Robert L. Garcea, and Pamela J. Bjorkman

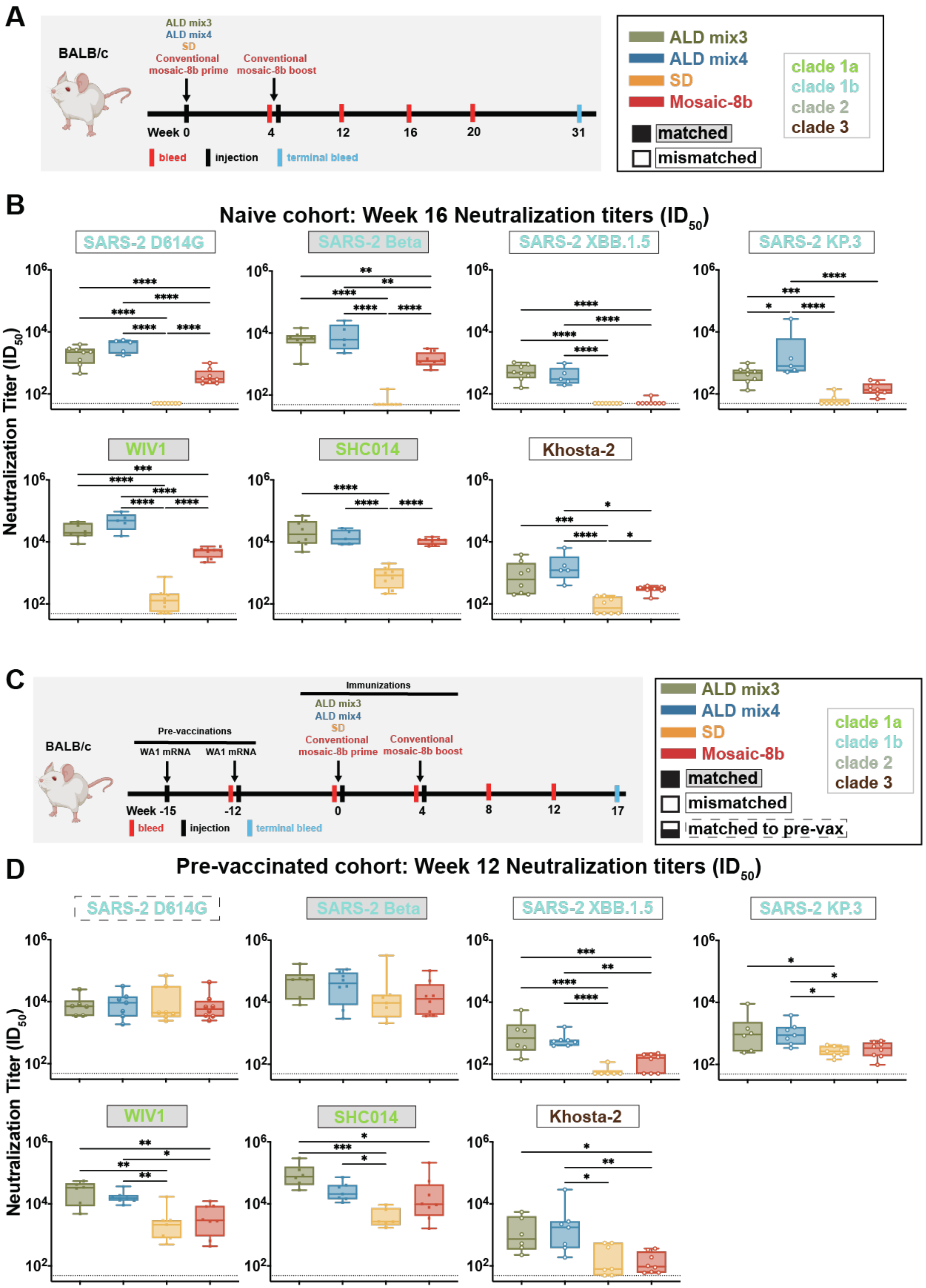
A
BALB/c
ALD mix3
ALD mix4
SD
Conventional mosaic-8b prime
Conventional mosaic-8b boost
Week 0
4
12
16
20
31
bleed
injection
terminal bleed
ALD mix3
ALD mix4
SD
Mosaic-8b
matched
mismatched
clade 1a
clade 1b
clade 2
clade 3
B
Naive cohort: Week 16 Neutralization titers (ID50)
SARS-2 D614G
SARS-2 Beta
SARS-2 XBB.1.5
SARS-2 KP.3
WIV1
SHC014
Khosta-2
Neutralization Titer (ID50)
C
BALB/c
Pre-vaccinations
WA1 mRNA
WA1 mRNA
Immunizations
ALD mix3
ALD mix4
SD
Conventional mosaic-8b prime
Conventional mosaic-8b boost
Week -15
-12
0
4
8
12
17
bleed
injection
terminal bleed
ALD mix3
ALD mix4
SD
Mosaic-8b
matched
mismatched
matched to pre-vax
clade 1a
clade 1b
clade 2
clade 3
D
Pre-vaccinated cohort: Week 12 Neutralization titers (ID50)
SARS-2 D614G
SARS-2 Beta
SARS-2 XBB.1.5
SARS-2 KP.3
WIV1
SHC014
Khosta-2
Neutralization Titer (ID50)

Figure S1. ALD-coated mosaic-8b immunization elicits more broadly matched and mismatched neutralizing Ab responses than conventional mosaic-8b immunizations in naïve and pre-vaccinated mice. Significant differences between cohorts linked by vertical lines in panels B and C are indicated by asterisks: p<0.05 = *, p<0.01 = **, p<0.001 = ***, p<0.0001 = ****. (A) Left: Schematic of immunization regimen for originally naïve mice. Mice were injected at week 0 with mosaic-8b RBD-nanoparticles administered as ALD mix3, ALD mix4, SD, or conventionally (bolus injection with adjuvant). At week 4, conventionally immunized mice were given an additional bolus injection of mosaic-8b RBD-nanoparticles plus adjuvant. Right: Colors used to identify immunizations and symbols used to identify matched (filled in square data points; gray shading around name) or mismatched (unfilled square data points; black outline around name) sarbecovirus antigens. Colors used throughout the figure indicate clades of sarbecovirus strains. (B) Neutralization potencies for serum samples from week 16 after immunization presented as half-maximal inhibitory dilutions (ID_{50} values) of sera against pseudoviruses from the indicated coronavirus strains (results for weeks 12 and 31 are shown in Figure 2). Dashed horizontal lines correspond to the limit of detection. Data for each immunization group are visualized using box and whisker plots, with each data point representing serum from one animal. The boxes display the range between the upper and lower quartiles, with a line denoting the median value. The whiskers extend to minimum and maximum values, excluding any outliers. Significantly higher neutralization titers were found for the following pairwise comparisons: against SARS-2 D614G (ALD mix3 vs. Mosaic-8b p<0.0001, ALD mix3 vs. SD p<0.0001, ALD mix4 vs. Mosaic-8b p<0.0001, ALD mix4 vs. SD p<0.0001), SARS-2 Beta (ALD mix3 vs. Mosaic-8b p=0.0014, ALD mix3 vs. SD p<0.0001, ALD mix4 vs. Mosaic-8b 8b p=0.0011, ALD mix4 vs. SD p<0.0001), SARS-2 XBB.1.5 (ALD mix3 vs. Mosaic-8b p<0.0001, ALD mix3 vs. SD p<0.0001, ALD mix4 vs. Mosaic-8b p<0.0001, ALD mix4 vs. SD p<0.0001), SARS-2 KP.3 (ALD mix3 vs. SD p=0.0003. ALD mix3 vs. ALD mix4 p=0.0326, ALD mix4 vs. Mosaic-8b p<0.0001, ALD mix4 vs. SD p<0.0001), WIV1 (ALD mix3 vs. Mosaic-8b p=0.0004, ALD mix3 vs. SD p<0.0001, ALD mix4 vs. Mosaic-8b p<0.0001, ALD mix4 vs. SD p<0.0001), SHC014 (ALD mix3 vs. SD p<0.0001, ALD mix4 vs. SD p<0.0001), and Khosta-2 (ALD mix3 vs. SD p=0.0003, ALD mix4 vs. Mosaic-8b p=0.0145, ALD mix4 vs. SD p<0.0001) (C) Left: Schematic of vaccination/immunization regimen for pre-vaccinated mice. Mice were vaccinated twice (3 weeks apart) with mRNA-LNP vaccines encoding the WA1 spike. At week 0 (15 weeks after the first vaccination), mice were injected with mosaic-8b RBD-nanoparticles administered as ALD mix3, ALD mix4, SD, or conventionally (bolus injection with adjuvant). At week 4, conventionally immunized mice were given an additional bolus injection of mosaic-8b RBD-nanoparticles plus adjuvant. Right: Right: Colors used to identify immunizations and symbols used to identify matched (filled in square data points; gray shading around name), matched to the WA1 pre-vaccination (half-filled in square data points; dashed black outline around name), or mismatched (unfilled square data points; black outline around name) sarbecovirus antigens. (D) Neutralization potencies for serum samples from week 12 after immunization presented as half-maximal inhibitory dilutions (ID_{50} values) of sera against pseudoviruses from the indicated coronavirus strains (results for weeks 8 and 17 are shown in Figure 3). Data for each immunization group are visualized using box and whisker plots, with each data point representing serum from one animal. The boxes display the range between the upper and lower quartiles, with a line denoting the median value. The whiskers extend to minimum and maximum values, excluding any outliers. Significantly higher neutralization titers were found for the following pairwise combinations: SARS-2 XBB.1.5 (ALD mix3 vs. Mosaic-8b p=0.0008, ALD mix3 vs. SD p<0.0001, ALD mix4

vs. Mosaic-8b p=0.0022, ALD mix4 vs. SD p<0.0001), SARS-2 KP.3 (ALD mix3 vs. SD p=0.0411, ALD mix4 vs. Mosaic-8b p=0.0474, ALD mix4 vs. SD p=0.0314), WIV1 (ALD mix3 vs. Mosaic-8b p=0.0048, ALD mix3 vs. SD p=0.0010, ALD mix4 vs. Mosaic-8b p=0.0159, ALD mix4 vs. SD p=0.0033), SHC014 (ALD mix3 vs. Mosaic-8b p=0.0172, ALD mix3 vs. SD p=0.001, ALD mix4 vs. SD p=0.0123), and Khosta-2 (ALD mix3 vs. Mosaic-8b p=0.0203, ALD mix4 vs. Mosaic-8b p=0.0039, ALD mix4 vs. SD p=0.0115).

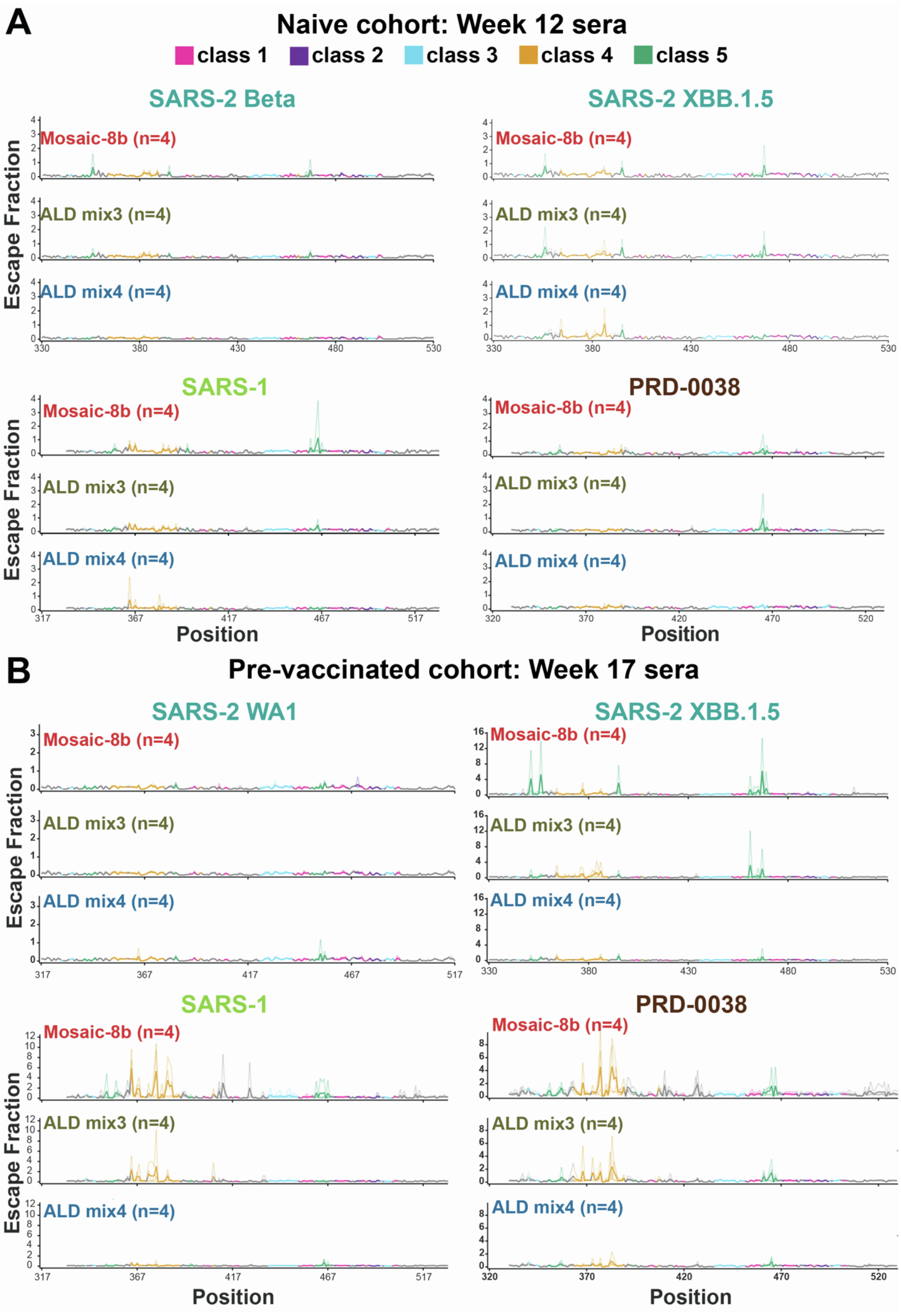
A
Naive cohort: Week 12 sera
class 1
class 2
class 3
class 4
class 5
SARS-2 Beta
SARS-2 XBB.1.5
SARS-1
PRD-0038
Mosaic-8b (n=4)
ALD mix3 (n=4)
ALD mix4 (n=4)
Escape Fraction
Position
B
Pre-vaccinated cohort: Week 17 sera
SARS-2 WA1
SARS-2 XBB.1.5
SARS-1
PRD-0038
Mosaic-8b (n=4)
ALD mix3 (n=4)
ALD mix4 (n=4)
Escape Fraction
Position

Figure S2. DMS line plots (same data are plotted on RBD structures in Figure 4). (A,B) Line plots for DMS results from the indicated number of samples for the RBD libraries listed at the top of each set of three plots. Mice were immunized with the immunogens indicated above each line plot. X-axis: RBD residue number. Y-axis: sum of the Ab escape of all mutations at a site (larger numbers = more Ab escape). Each line represents one antiserum with heavy lines showing the average across the n=4 sera in each group. Lines are colored according to RBD epitopes in Figure 4A.

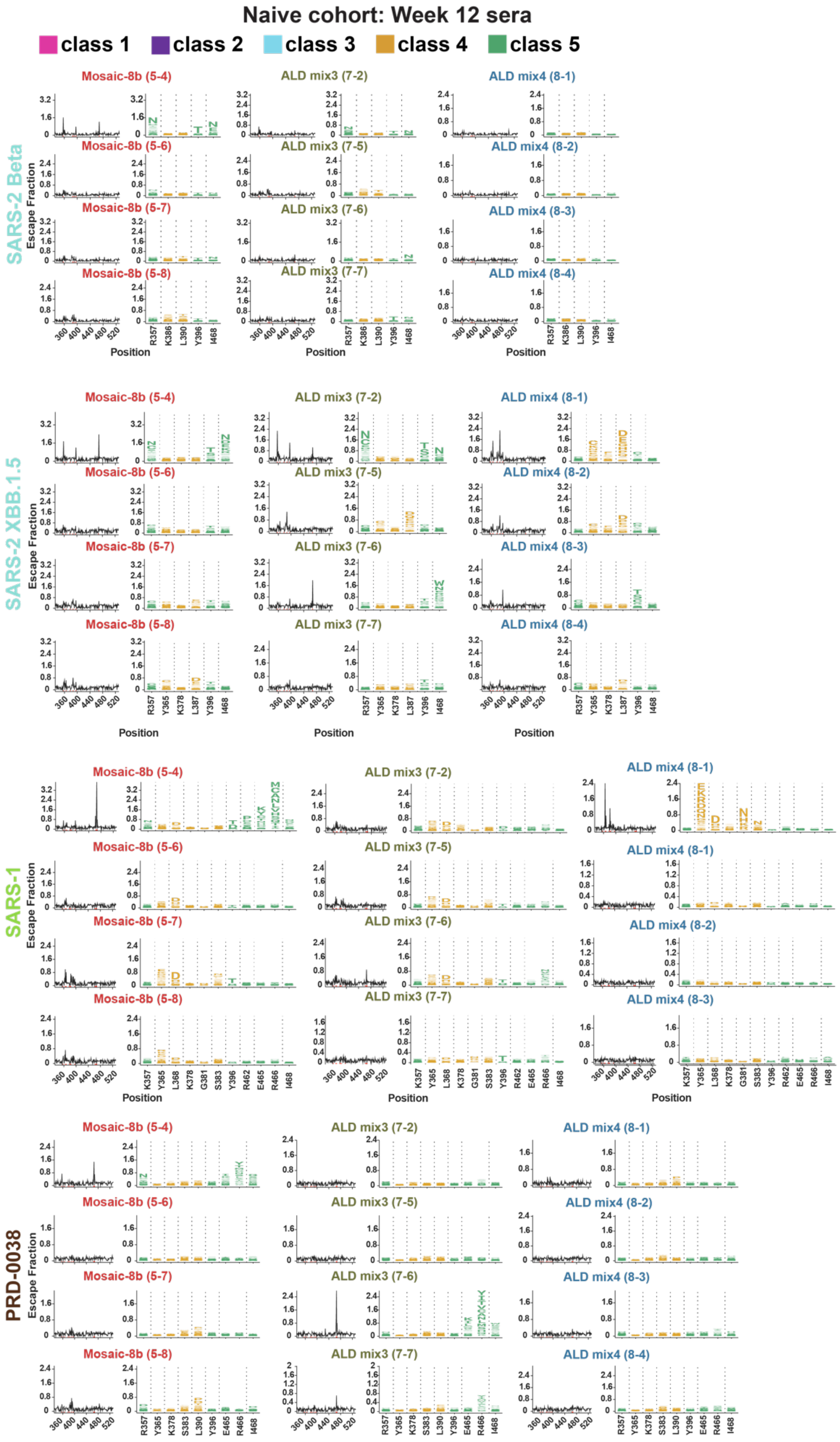

Naive cohort: Week 12 sera
class 1
class 2
class 3
class 4
class 5
SARS-2 Beta
SARS-2 XBB.1.5
SARS-1
PRD-0038
Escape Fraction
Position
Mosaic-8b (5-4)
Mosaic-8b (5-6)
Mosaic-8b (5-7)
Mosaic-8b (5-8)
ALD mix3 (7-2)
ALD mix3 (7-5)
ALD mix3 (7-6)
ALD mix3 (7-7)
ALD mix4 (8-1)
ALD mix4 (8-2)
ALD mix4 (8-3)
ALD mix4 (8-4)

Figure S3. DMS line and logo plots for individual originally naïve mice. Line (left) and logo plots (right) are shown for DMS results for sera from individual mice (IDs in parentheses) collected at 12 weeks after immunization with either mosaic-8b, ALD mix3, or ALD mix4 (immunization schedule in Figure 2A). DMS was performed using the indicated RBD libraries. X-axes of line and logo plots show RBD residue numbers. Y-axes of line plots show the sum of the Ab escape of all mutations at a site (larger numbers indicate increased Ab escape). Sites with the strongest Ab escape are shown in logo plots, with tall letters representing the most frequent mutations at a site. Logo plots are colored for RBD epitopes within different classes[1-4] (class 1 = pink; class 2 = purple; class 3 = blue; class 4 = yellow; class 5 = green; epitopes are shown in Figures 1E and 4A; gray for residues not assigned to an epitope). Compiled data from all mice in each group are plotted on RBD structures in Figure 4 and as line plots in Figure S2.

Pre-vaccinated cohort: Week 17 sera

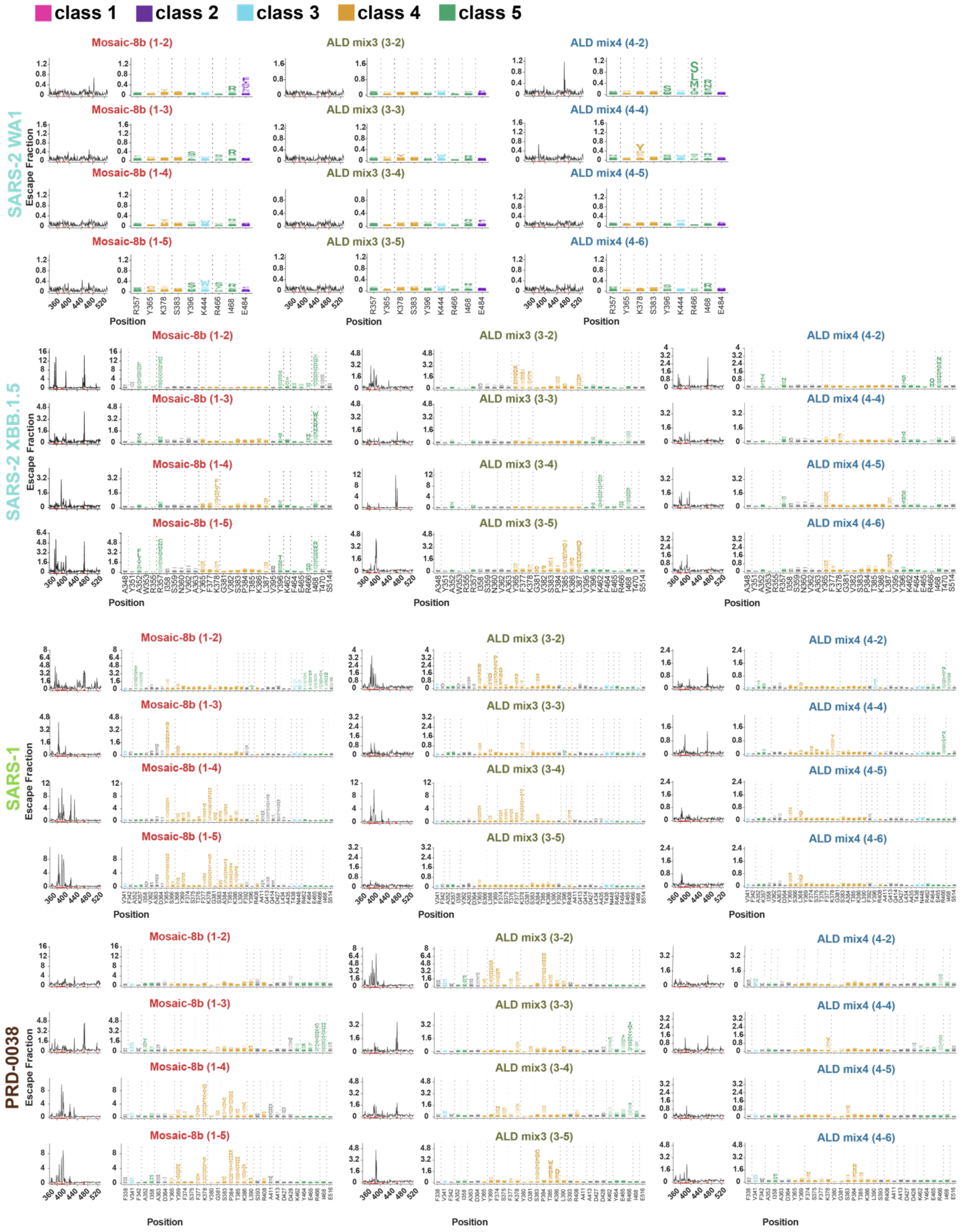

Figure S4. DMS line and logo plots for individual pre-vaccinated mice. Line (left) and logo plots (right) are shown for DMS results at week 17 for sera from individual mice (IDs in parentheses) that were pre-vaccinated with two doses of WA1 mRNA-LNP and then immunized with either mosaic-8b, ALD mix3, or ALD mix4 (vaccination/immunization schedule in Figure 3A). DMS was performed using the indicated RBD libraries. X-axes of line and logo plots show RBD residue numbers. Y-axes of line plots show the sum of the Ab escape of all mutations at a site (larger numbers indicate increased Ab escape). Sites with the strongest Ab escape are shown in logo plots, with tall letters representing the most frequent mutations at a site. Logo plots are colored differently for RBD epitopes within different classes[1-4] (class 1 = pink; class 2 = purple; class 3 = blue; class 4 = yellow; class 5 = green; epitopes are shown in Figures 1E and 4A; gray for residues not assigned to an epitope). Compiled data from all mice in each group are plotted on RBD structures in Figure 4 and as line plots in Figure S2.

References

1 Barnes, C. O., Jette, C. A., Abernathy, M. E., Dam, K.-M. A., Esswein, S. R., Gristick, H. B., Malyutin, A. G., Sharaf, N. G., Huey-Tubman, K. E., Lee, Y. E., Robbiani, D. F., Nussenzweig, M. C., West, A. P. & Bjorkman, P. J. (2020). SARS-CoV-2 neutralizing antibody structures inform therapeutic strategies. Nature *588*, 682–687.

2 Jette, C. A., Cohen, A. A., Gnanapragasam, P. N. P., Muecksch, F., Lee, Y. E., Huey-Tubman, K. E., Schmidt, F., Hatziioannou, T., Bieniasz, P. D., Nussenzweig, M. C., West, A. P., Keeffe, J. R., Bjorkman, P. J. & Barnes, C. O. (2021). Broad cross-reactivity across sarbecoviruses exhibited by a subset of COVID-19 donor-derived neutralizing antibodies. Cell reports *36*, 109760.

3 Jensen, J. L., Sankhala, R. S., Dussupt, V., Bai, H., Hajduczki, A., Lal, K. G., Chang, W. C., Martinez, E. J., Peterson, C. E., Golub, E. S., Rees, P. A., Mendez-Rivera, L., Zemil, M., Kavusak, E., Mayer, S. V., Wieczorek, L., Kannan, S., Doranz, B. J., Davidson, E., Yang, E. S., Zhang, Y., Chen, M., Choe, M., Wang, L., Gromowski, G. D., Koup, R. A., Michael, N. L., Polonis, V. R., Rolland, M., Modjarrad, K., Krebs, S. J. & Joyce, M. G. (2023). Targeting the Spike Receptor Binding Domain Class V Cryptic Epitope by an Antibody with Pan-Sarbecovirus Activity. J Virol *97*, e0159622.

4 Cui, L., Li, T., Lan, M., Zhou, M., Xue, W., Zhang, S., Wang, H., Hong, M., Zhang, Y., Yuan, L., Sun, H., Ye, J., Zheng, Q., Guan, Y., Gu, Y., Xia, N. & Li, S. (2024). A cryptic site in class 5 epitope of SARS-CoV-2 RBD maintains highly conservation across natural isolates. iScience *27*, 110208.
